# Cytokine Expression Profiling in Idiopathic Pulmonary Fibrosis: Insights From Integrative Proteomic Analysis

**DOI:** 10.1155/carj/2272156

**Published:** 2025-11-07

**Authors:** Chenyou Shen, Wei Wang, Guirong Li, Dong Wei, Xusheng Yang, Cheng Jiang, Yating Sheng, Yuan Chen, Jingjing Xu, Shugao Ye, Jingyu Chen

**Affiliations:** ^1^Department of Lung Transplantation, The Affiliated Wuxi People's Hospital of Nanjing Medical University, Wuxi People's Hospital, Wuxi Medical Center, Nanjing Medical University, 299 Qingyang Road, Wuxi 214023, Jiangsu, China; ^2^Department of Anesthesiology, The Affiliated Wuxi People's Hospital of Nanjing Medical University, Wuxi People's Hospital, Wuxi Medical Center, Nanjing Medical University, 299 Qingyang Road, Wuxi 214023, Jiangsu, China

**Keywords:** bioinformatics analysis, cytokines, idiopathic pulmonary fibrosis, potential drugs, protein microarray

## Abstract

**Introduction:**

Idiopathic pulmonary fibrosis (IPF) is a chronic progressive fibrotic lung disease with a poor prognosis and no effective pharmacological treatments. Cytokines are a class of small-molecule proteins with diverse biological activities. Many cytokines—most notably transforming growth factor β—have been demonstrated to play an important role in IPF. However, a few studies have systematically described the relationship between cytokines and IPF.

**Methods:**

Lung tissues from controls and patients with IPF were collected during lung transplantation. The expression profiles of 440 cytokines in lung tissues were obtained using protein microarrays. Proteomic analysis was performed, and differentially expressed proteins (DEPs) were identified. Furthermore, an integrative bioinformatics analysis was performed and included functional enrichment analysis, protein–protein interaction (PPI) network construction, hub protein determination, immune cell infiltration analysis, potential drug prediction, and single-cell analysis. The hub protein expression was validated through Gene Expression Omnibus (GEO) database evaluation and immunochemical analysis.

**Results:**

32 DEPs were identified from the two groups. They were mainly enriched in cell chemotaxis, basal part of cell, and growth factor binding and were involved in PI3K–Akt signaling. The PPI network was constructed for the DEPs, and five hub proteins (FGF2, HGF, HBEGF, ERBB3, and ANGPT2) were identified. The immune infiltration analysis demonstrated a significantly higher percentage of resting NK cells in IPF lung tissue. The drug prediction analyses identified 13 potential candidates targeting the five hub proteins. The single-cell analysis predicted the cellular localization of each key cytokine.

**Conclusions:**

Using protein microarrays, we obtained comprehensive cytokine expression profiles in control and IPF lung tissues and conducted an integrated bioinformatics analysis of the proteomic data. Our findings may improve the comprehension of the role of cytokines in IPF and the underlying mechanisms. Moreover, they provide novel targets for developing safe and efficacious drugs for treating IPF.

## 1. Introduction

Idiopathic pulmonary fibrosis (IPF) represents a more common variety of prolonged interstitial lung disease (ILD) of unknown etiology. IPF is categorized with the umbrella term idiopathic interstitial pneumonias, with pathological manifestations of usual interstitial pneumonia. IPF onset is insidious, with symptoms developing after the age of 50 in most cases. The principal clinical manifestation is dyspnea, frequently accompanied by a dry cough. Lung imaging typically reveals diffuse grid-like and honeycomb shadows in the basal and peripheral areas of both lungs, whereas lung function testing demonstrates restrictive ventilatory deficits. The disease is typically progressive, ultimately resulting in respiratory failure and death [1, 2]. The IPF etiology is still unclear. However, much evidence points to a potential association with genetic predisposition, tobacco consumption, environmental factors, viral infections, gastroesophageal reflux, and other risk factors [3, 4]. Considering the ineffectiveness of antifibrotic drug therapy, IPF generally has a poor prognosis, where median survival is only 2–3 years following diagnosis. Lung transplantation represents the only effective cure for IPF; however, this therapy is relevant to only a few patients, taking into account their age, economic status, and comorbidities [5, 6]. Therefore, identifying safe and efficacious drug targets for this fatal disease is extremely important.

Cytokines are small-molecule proteins whose synthesis and secretion occur in response to stimulation by various immune cells, for example, macrophages, monocytes, T and B cells, and natural killer (NK) cells, and specific nonimmune cells (epidermal cells, endothelial cells, and fibroblasts). Cytokines regulate wide-ranging biological functions through cell–cell interactions, facilitating information transfer between cells. In physiological states, cytokines are pivotal in sustaining homeostasis and boosting the repair and regeneration of tissue. Nevertheless, aberrant expression and secretion of cytokines in specific pathological conditions, for example, inflammation, infection, and tumor, might result in a disease developing and progressing [7–9].

Substantial evidence has demonstrated that cytokines are key in IPF pathogenesis. For example, transforming growth factor β (TGF-β) is a central mediator in pulmonary fibrosis development and progression and exerts a multitude of biological effects, including inflammatory cell recruitment, extracellular matrix accumulation, promotion of wound repair, and induction of fibroblast activation [10, 11]. The profibrotic cytokine connective tissue growth factor (CTGF) exerts its effects by inducing fibroblast-to-myofibroblast transformation and epithelial–mesenchymal transition and working with other profibrotic factors, for example, TGF-β [12, 13]. In contrast, interferon-γ promotes the formation of an antifibrotic immune microenvironment in the lung by, among other things, inhibiting TGF-β activity, thereby alleviating the progression of pulmonary fibrosis [14, 15]. Furthermore, on the therapeutic front, cytokine-targeted interventions have become a key strategy in IPF treatment. For example, nintedanib and pirfenidone, which are the pharmaceutical agents currently approved by the FDA regarding IPF clinical treatment, inhibit various profibrotic cytokines or their receptors, thereby reducing fibroblast proliferation and extracellular matrix deposition [16–18].

Because of the crucial role of cytokines in IPF and their potential value in IPF treatment, there has been a sustained interest in the relationship between cytokines and IPF in recent years. However, most existing studies have centered on a specific cytokine or group of cytokines in IPF. A few studies have systematically examined the link between cytokines and IPF development. Therefore, we used protein microarrays to more comprehensively determine the expression of 440 cytokines related to important biological processes (BPs)—including immune response, chemotaxis, differentiation, and angiogenesis—in the lung tissues from donors and patients with IPF. We identified proteins that were differentially expressed; analyzed their functions, interactions, and immune infiltration; predicted potential drugs; and validated the expression of hub proteins. Overall, the present study yields new insights regarding the cytokine expression profiles in IPF lung tissues. The findings may offer novel perspectives on the IPF pathogenesis and suggest possible drug targets for treating IPF.

## 2. Methods

### 2.1. Patients and Lung Tissue Samples

Fresh lung tissues were obtained from five patients with IPF who had undergone lung transplantation in April 2023 at the Affiliated Wuxi People's Hospital of Nanjing Medical University (Jiangsu, China). IPF diagnosis was based on the American Thoracic Society/European Respiratory Society/Japanese Respiratory Society/Asociación Latinoamericana de Tórax (ATS/ERS/JRS/ALAT) guideline published in 2022 [19]. Patients with ILDs with known causes (pneumoconiosis, ILD associated with connective tissue disease, and lung disease induced by drugs) were excluded from the study. The control group comprised lung tissues obtained from five healthy donors following volume reduction during lung transplantation in the same period. For each case, the isolated lung tissue was divided into two parts: One part was rapidly frozen in liquid nitrogen and stored at −80°C, while the other was fixed in 4% paraformaldehyde and embedded in a paraffin block. The Institutional Research Ethics Committee of Wuxi People's Hospital approved the human sample collection protocols (2023-126). Informed consent forms were signed by the patients and donors included in the study or their family members.

### 2.2. Protein Microarray

The IPF and control lung tissues were completely lysed with a protease inhibitor cocktail (Cell Signaling Technology, USA) and cell lysis buffer (RayBiotech, USA) at low temperature to obtain tissue lysates. Subsequently, the BCA method was used to establish the protein concentration in the lysate supernatant. Thereafter, the protein microarray was performed following the instructions of the manufacturer (RayBiotech, catalogue number: GSH-CAA-440) to establish the expression profiles of cytokines in the samples. The protein microarray is based on the double-antibody sandwich method, which facilitates the determination of the relative expression of 440 cytokines in human samples. The Cy3 channel signals were scanned using a laser scanner (InnoScan 300 Microarray Scanner, France). Raw numerical data from the array scan were analyzed using the RayBio Analysis Tool (catalogue number: GSH-CAA-440-SW).

### 2.3. Identification of Differentially Expressed Proteins (DEPs)

Box plots were used to visualize the data distribution for each sample. The analysis was conducted in R 4.2.1 and visualized with R ggplot2 (Version 3.3.6). We used principal component analysis (PCA) to determine the similarities and differences between the IPF and control groups. This was achieved by extracting feature components from high-dimensional data, converting them to low-dimensional data, and displaying these features in a two-dimensional plot. The PCA was conducted in R and visualized using the R ggplot2 package (Version 3.3.6). The DEP screening threshold was fixed at fold change > 1.2 or < 0.83, with an adjusted *p* < 0.05. We generated a volcano plot using R ggplot2. A DEP expression heatmap was constructed with R ComplexHeatmap.

### 2.4. Functional Enrichment Analysis

We conducted the Gene Ontology (GO) annotation and Kyoto Encyclopedia of Genes and Genomes (KEGG) pathway enrichment analyses of the DEPs with R clusterProfiler (Version 4.4.4). The GO annotations were cellular component (CC), BP, and molecular function (MF) [20]. The enrichment analysis results were visualized using R ggplot2 (Version 3.3.6). The significance cutoff value was fixed at an adjusted *p* value of < 0.05.

Furthermore, to analyze the enrichment pertaining to BPs within the entire set of 440 cytokines included in the microarray, the “c5.go.bp.v2022.1.Hs.symbols.gmt” gene set was obtained from MSigDB (https://www.gsea-msigdb.org/gsea/msigdb), and gene set enrichment analysis (GSEA) was conducted using R clusterProfiler (Version 4.4.4). Unlike traditional enrichment analysis, which uses a fixed threshold to analyze only differentially expressed genes or DEPs, GSEA analyzes all detected genes or proteins using a predefined gene set, regardless of the threshold. Hence, GSEA can detect subtle, ordered changes in BPs or pathways that traditional methods might miss [21]. The GSEA results were visualized using R ggplot2 (Version 3.3.6). An FDR < 0.25 and adjusted *p* < 0.05 indicated that enrichment was considered significant.

### 2.5. Construction of Protein–Protein Interaction (PPI) Network and Identification of Hub Proteins

The DEP interactions were analyzed, and the resulting network was constructed using Search Tool for the Retrieval of Interacting Genes/Proteins (STRING, Version 12.0, https://string-db.org/). The minimum required interaction score was set to 0.4. Moreover, the Cytoscape (Version 3.9.1) cytoHubba plugin was utilized to calculate the protein scores using the maximal clique centrality (MCC) algorithm. The proteins with the top five MCC scores were subsequently identified as hub proteins. The Friends analysis was performed with R GOSemSim (Version 2.22.0) to calculate the similarity between the hub proteins and rank them accordingly [22–24].

### 2.6. Prediction of the Interaction Networks of Hub Proteins and Transcription Factors

The potential relationships between the hub proteins and the molecules with which they may interact were further predicted using the GeneMania database (https://genemania.org/). The Transcriptional Regulatory Relationships Unraveled by Sentence-based Text (TRRUST mining, Version 2.0, https://www.grnpedia.org/trrust/) database was utilized to predict the transcriptional regulatory network of the hub proteins, and the results were visualized using NetworkAnalyst (https://www.networkanalyst.ca/) [25, 26].

### 2.7. Immune Infiltration Analysis

Based on the CIBERSORT core algorithm, markers for 22 immune cells provided on the CIBERSORTx website (https://cibersortx.stanford.edu/) were utilized to quantify the immune cell infiltration in each protein microarray sample [27]. A Spearman correlation analysis was conducted to elucidate the interrelationships between the various immune cells. The resulting data were subsequently visualized using the R ggplot2 package (Version 3.3.6). Violin plots were constructed to determine the composition of the 22 immune cells in the two groups using R ggplot2 (Version 3.3.6). For immune cells that demonstrated significant compositional differences between the two groups, correlation lollipop plots were constructed to depict the link between the significantly different immune cells and the hub proteins using R ggplot2 (Version 3.3.6).

### 2.8. Potential Drug Prediction

The Drug–Gene Interaction Database (DGIdb, Version 4.0, https://www.dgidb.org/) supplies data on drug–gene interactions and druggable genes derived from a range of reliable sources, including publications and databases [28]. Potential target drugs against the five hub proteins were retrieved from DGIdb, and the results were downloaded and imported into Cytoscape (Version 3.9.1) to map the drug–hub protein potential interaction network.

Furthermore, the potential protective effect and clinical translation value of the predicted drugs for treating pulmonary fibrosis were explored through a literature review. The literature review was conducted by systematically searching the PubMed database for each candidate using the drug name and “pulmonary fibrosis” as keywords.

### 2.9. GEO Database Validation of the Hub Proteins

Hub protein expression was validated using three GEO datasets: GSE24206, GSE53845, and GSE92592. The GSE24206 dataset contains the gene expression profiles of lung tissue samples from 11 IPF patients and six healthy controls, as determined with the Affymetrix Human Genome U133 Plus 2.0 Array (platform: GPL570). The GSE53845 dataset contains the gene expression profiles of lung tissue samples from 40 IPF patients and eight healthy controls, as determined with the Agilent-014850 Whole Human Genome Microarray (platform: GPL6480). Lastly, the GSE92592 dataset includes the gene expression profiles of lung tissue samples from 20 IPF patients and 19 controls, as determined with the Illumina HiSeq 2000 (platform: GPL11154). The gene expression profile data were analyzed using the GEO2R web tool, and the results were visualized using GraphPad Prism 8. Each dataset was processed and analyzed independently.

### 2.10. Immunohistochemical Validation of the Hub Proteins

Immunohistochemical analysis was performed on the aforementioned lung tissue samples (control: 5, IPF: 5) to verify the expression of key cytokines at the protein level. Lung tissue wax blocks were cut into 5-μm-thick sections, which were then deparaffinized, rehydrated, and processed for antigen retrieval, blocking, and primary antibody incubation at 4°C overnight. The following primary antibodies were used: rabbit anti-FGF2 (1:200; Proteintech, USA, 11234-1-AP), rabbit anti-HGF (1:200; Proteintech, 26881-1-AP), goat anti-HBEGF (15 μg/mL; R&D Systems, USA, AF-259-NA), rabbit anti-ERBB3 (1:200; Proteintech, 10369-1-AP), and goat anti-ANGPT2 (15 μg/mL; R&D Systems, AF623). After washing, the sections were incubated with the corresponding secondary antibodies, followed by DAB chromogenic detection. Then, the sections were counterstained with hematoxylin and visualized under an Olympus light microscope. The hub protein expression underwent quantification analysis using Fiji (ImageJ).

### 2.11. Single-Cell Analysis

We explored the cellular localization of hub cytokines at the single-cell level using the GEO dataset GSE136831, which includes single-cell RNA sequencing data from the lungs of 32 IPF patients and 28 controls. The cells were annotated using a marker gene set from a previous study, where the GSE136831 dataset originated [29]. The single-cell annotation information, and other integrated data, was downloaded from the GSE136831 dataset (https://www.ncbi.nlm.nih.gov/geo/query/acc.cgi?acc=GSE136831). A standardized analysis containing data quality control (percent.mt < 10 and nCount_RNA > 200), normalization, identification of high-variance genes, PCA, and t-distributed stochastic neighbor embedding (t-SNE) dimensionality reduction clustering was performed using the R package Seurat (Version 4.4.0). Next, the data were systematically analyzed and visualized using the R package scCustomize (Version 3.0.1). The t-SNE subgroup mapping was constructed using the DimPlot_scCustom function to illustrate the overall distribution of cell subpopulations. The expression of the key cytokines in each cell subpopulation was revealed using the VlnPlot_scCustom function to demonstrate their individual expression characteristics. The expression patterns of key cytokines in t-SNE space were visualized using the FeaturePlot_scCustom function. Furthermore, single-cell expression of the hub cytokines was analyzed using the FindMarkers function in Seurat. The expression levels of the hub genes within each cell population in the control and IPF groups were analyzed. The significance of the comparisons was evaluated using a Wilcoxon rank sum test, and the *p* values were corrected using the Benjamini–Hochberg adjustment.

### 2.12. Statistical Analysis

Statistical analyses were performed using R 4.2.1 and GraphPad Prism 8. All data conformed to the normal distribution assumption and are presented as the mean ± standard deviation. Two-tailed *t*-tests were used for comparisons between two groups, and *p* < 0.05 was considered statistically significant.

## 3. Results

### 3.1. Baseline Characteristics

**Figure 1:**
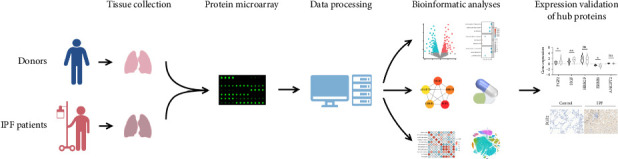
Overall study design flowchart.

Figure 1 presents the flowchart depicting the overall study design. The lung tissue samples were sourced from five patients with IPF (female: 1, male: 4; mean age: 62.8 ± 6.7 years, age range: 52–69 years) and five healthy donors (female: 1, male: 4; mean age: 39 ± 15.2 years, age range: 23–58 years). The patients' lung function was analyzed, with a predicted forced vital capacity percentage (FVC%) of 54.8 ± 17.1, a predicted diffusing capacity of the lung for carbon monoxide percentage (DLCO%) of 17.6 ± 12.7, and a 6-min walk test (6MWT) of 177 ± 80.7 m.  Table 1 presents the participants' clinical characteristics.

### 3.2. Identification of DEPs

**Figure 2:**
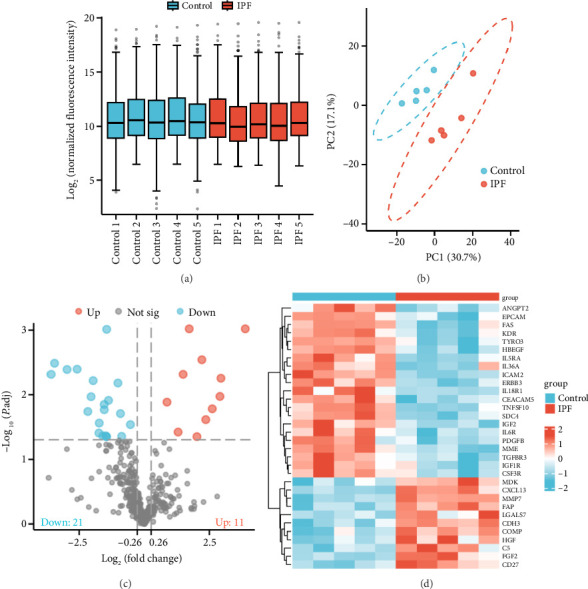
Identification of differentially expressed proteins (DEPs) between control individuals and patients with idiopathic pulmonary fibrosis (IPF). (a) Box plots of the overall distribution of cytokine expression across the 10 samples. *Y*-axis: The log_2_-transformed fluorescence intensity of each sample. Raw data were normalized to the median intensity of positive controls (POS1/POS2) with background subtraction, followed by log_2_ transformation; (b) principal component analysis of control and IPF samples; (c) volcano plot of cytokines detected in the microarray. The *x*-axis coordinates of 0.26 and −0.26 represent log_2_-transformed fold change values of 1.2 and 0.83, respectively, which are the screening thresholds for the differentially expressed proteins; (d) heatmap of the 32 DEPs identified between the control and IPF groups.

Box plots were used to analyze the overall characteristics of the data set. As illustrated in Figure 2(a), the distribution of data across samples in the microarray was found to be relatively consistent. The relationships between and within groups were subsequently analyzed by PCA. As shown in Figure 2(b), the samples from the IPF and control groups were observed to be clustered together, with samples from the same group clustered relatively close to each other, whereas samples from different groups were relatively distant from each other. This finding indicated that a distinction existed between the groups and that the data met the requisite criteria for further analysis.

The cytokine expression profiling data of the IPF and control groups were subjected to analysis with the screening threshold of fold change set at > 1.2 or < 0.83 and an adjusted *p* value set at < 0.05. The results demonstrated that a total of 32 proteins exhibited significant differential expression in the IPF group versus the control, with 11 proteins upregulated and 21 proteins downregulated (Figures 2(c), 2(d)). Additional file 1 (Table S1) contains the detailed information of the DEPs.

### 3.3. Functional Enrichment Analyses

**Figure 3:**
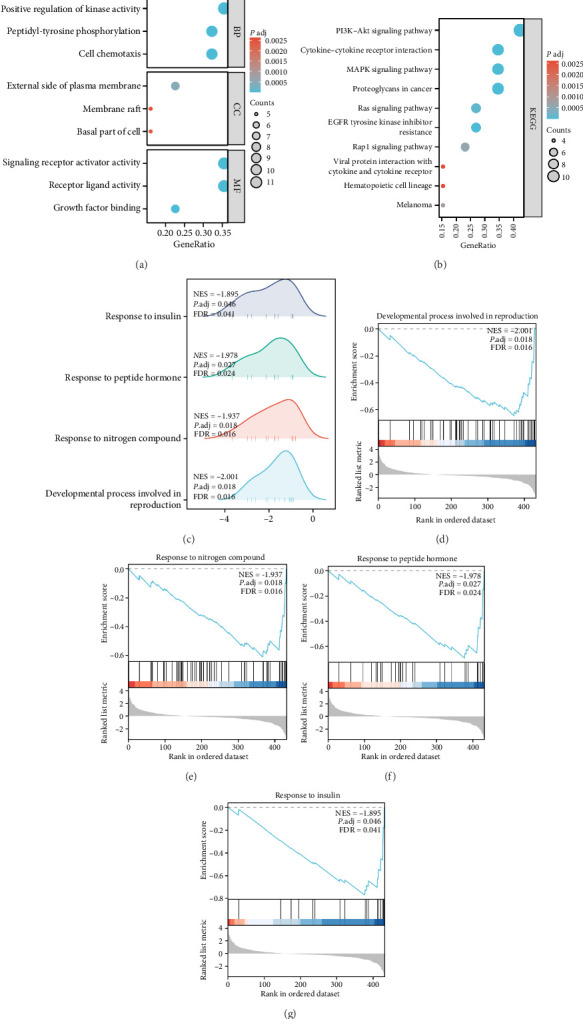
Functional enrichment analyses. (a) GO functional enrichment of differentially expressed proteins (DEPs); (b) KEGG pathway prediction for DEPs; (c) ridgeline plot depicting the four major biological processes identified through GSEA between the control and idiopathic pulmonary fibrosis (IPF) groups. *Y*-axis: The names of the gene sets. *X*-axis: The distribution of the log_2_ (fold change) values for core-enriched genes within each gene set; (d–g) the cytokines detected in the microarray demonstrated significant enrichment for biological processes, including (d) developmental process, involved in reproduction, (e) response to nitrogen compound, (f) peptide hormone response, and (g) insulin response.

The GO analysis of the DEPs demonstrated that they participated in numerous BPs, for example, positive regulation of kinase activity, peptidyl-tyrosine phosphorylation, and cell chemotaxis. Additionally, they were found to localize to CCs, which included the external region of the plasma membrane, membrane raft, and the basal part of the cell. Moreover, the DEPs were linked to various MFs such as activity of signaling receptor activators, activity of receptor ligands, and growth factor binding (Figure 3(a)). The KEGG signaling pathway prediction results indicated that the DEPs were closely associated with not only cytokine–cytokine receptor interaction but also the PI3K–Akt, MAPK, and Ras pathways and EGFR tyrosine kinase inhibitor resistance (Figure 3(b)). The details of the GO and KEGG items are presented in Table S2.

Further insight into the possible biological functions of the collection of cytokines detected by the microarray in IPF pathogenesis was obtained using GSEA. The core-enriched genes in each gene set were ranked by their relevance to the phenotype, which in this case was the log_2_ (fold change) value, and their distributional trends in significantly enriched BPs were illustrated with a ridgeline plot (Figure 3(c)). The results indicated that the 440 cytokines detected in the microarray were significantly enriched in a number of BPs, including the response to insulin, peptide hormone, and nitrogen compound, and developmental process, involved in reproduction (Figures 3(d), 3(e), 3(f), 3(g)). Details of the enrichment are shown in Table S3.

### 3.4. PPI Network Construction and Hub Protein Identification

**Figure 4:**
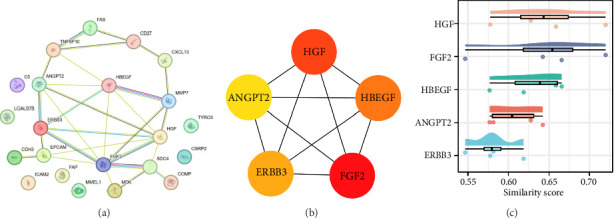
Construction of the protein–protein interaction (PPI) network and identification of hub proteins. (a) The PPI network of differentially expressed proteins (DEPs) generated from the STRING database; (b) top five hub proteins extracted from the PPI network using Cytoscape; (c) Cloud and rain plot reveals the relative importance of the five hub proteins. *X*-axis: Protein–protein similarity scores (range: 0–1), where higher values indicate stronger functional associations. Scores were calculated using GOSemSim.

The interaction relationship between DEPs was examined using a PPI network. As shown in Figure 4(a), a PPI network containing 22 nodes and 29 edges was generated based on the DEP analysis with the STRING database. Subsequently, the CytoHubba plugin was utilized to ascertain the top five hub proteins in the PPI network through the MCC algorithm. Five proteins with the highest scores among the 22 nodal proteins—FGF2, HGF, HBEGF, ERBB3, and ANGPT2, in descending order of scores—were identified as the hub proteins (Figure 4(b)). The Friends analysis demonstrated that, among the five hub proteins, HGF exhibited the greatest similarity to the other proteins, indicating that it is a key protein (Figure 4(c)).

### 3.5. Interaction and Transcriptional Regulatory Networks of Hub Proteins

**Figure 5:**
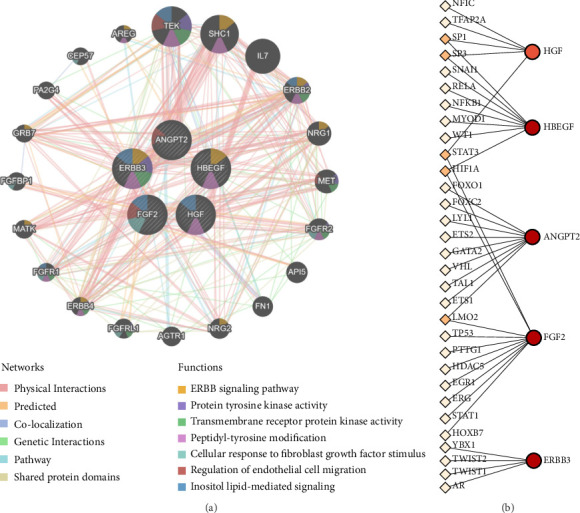
Interaction and transcriptional regulatory networks of hub proteins. (a) PPI network of the five hub proteins constructed with GeneMania. (b) Transcriptional regulatory network of genes that correspond to the hub proteins. The circles denote hub genes. The diamonds denote the predicted transcription factors, with the white diamonds indicating the regulation of a single hub gene and the orange diamonds indicating the regulation of two hub genes.

The GeneMania database was used to further predict the links between hub proteins and their potential interacting proteins, as well as the associated biological functions. As illustrated in Figure 5(a), the five central nodes representing hub proteins were circumscribed by 20 peripheral nodes, indicating that the proteins represented by the peripheral nodes have potential interconnections with the hub proteins. Among these forms of interconnections, physical interactions were the most prevalent, accounting for 84.31%. Furthermore, the primary functions and pathways associated with the hub protein network included the ERBB signaling pathway, protein tyrosine kinase activity, and transmembrane receptor protein kinase activity.

The transcription factors associated with hub genes were explored based on the corresponding genes encoding the hub proteins using the TRRUST database. Next, the hub gene transcriptional regulatory network was visualized using the NetworkAnalyst visual analysis tool. As illustrated in Figure 5(b), the network encompassed 31 transcription factors, among which the five transcription factors in orange (SP1, SP3, STAT3, HIF1A, LMO2) demonstrated the potential to regulate the transcription of two hub genes.

### 3.6. Immune Cell Infiltration Analysis

**Figure 6:**
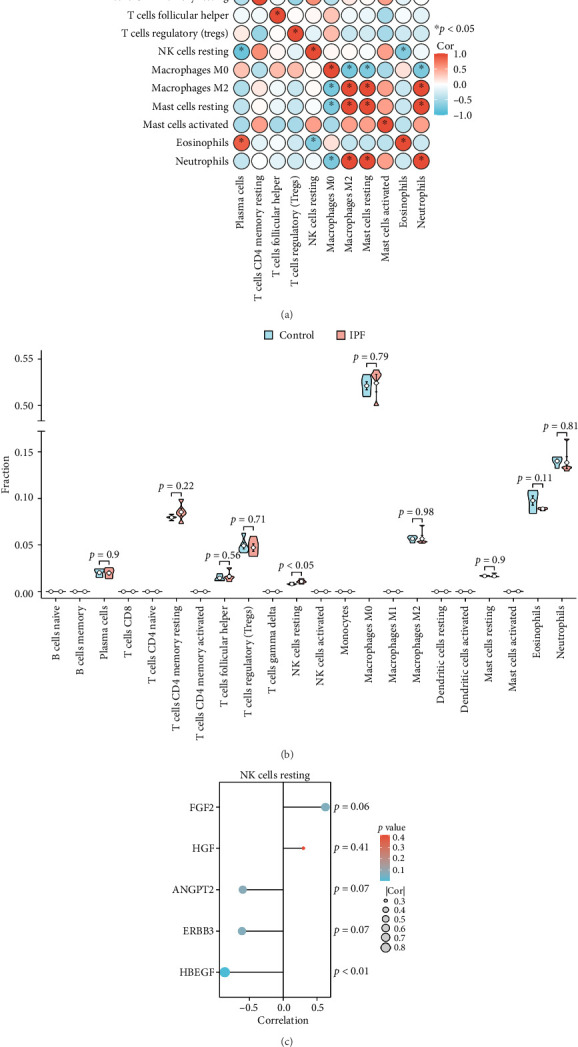
Immune cell infiltration analysis. (a) Correlation heatmap of 11 immune cell types with a composition percentage > 0. (b) Violin plots of immune cell infiltration in the control and idiopathic pulmonary fibrosis (IPF) groups. (c) Lollipop plots show the correlation between differentially infiltrated immune cells (i.e., resting natural killer [NK] cells) and hub proteins. HBEGF is significantly negatively correlated with the resting NK cell level (*r* = −0.87, *p*=0.0027). ^∗^*p* < 0.05.

Given the interrelated nature of cytokine synthesis, secretion, and biological function with the immune environment, we analyzed the infiltration of 22 immune cell types in lung tissue using CIBERSORT. Correlation analysis of 11 immune cell types with a composition percentage > 0 revealed significant positive correlations between plasma cells and eosinophils, between M2 macrophages and resting mast cells, between M2 macrophages and neutrophils, and between resting mast cells and neutrophils. In contrast, significant negative correlations were observed between plasma cells and resting NK cells, between resting NK cells and eosinophils, between M0 macrophages and M2 macrophages, between M0 macrophages and resting mast cells, and between M0 macrophages and neutrophils (Figure 6(a)). The violin plots (Figure 6(b)) demonstrate the diverse immune cell infiltration in IPF versus the control; the resting NK cell level in IPF lung tissue was markedly higher than that in the control lung tissue. We further examined the correlation between immune cells with significant infiltration differences—that is, resting NK cells—and hub proteins. As illustrated in Figure 6(c), of the five hub proteins, HBEGF was significantly negatively correlated with the resting NK cell level.

### 3.7. Potential Drug Prediction

**Figure 7:**
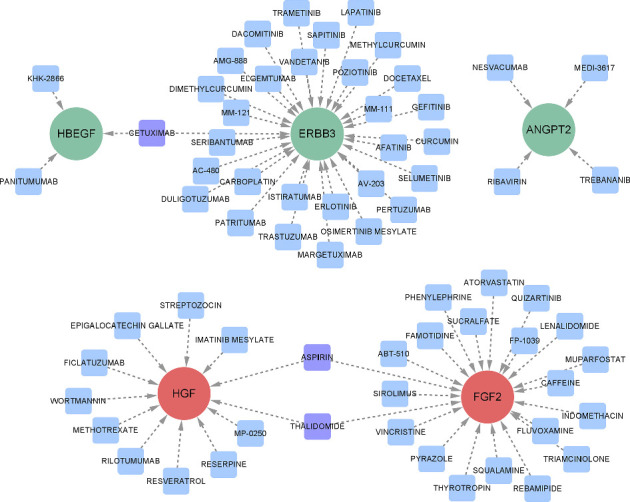
Drug–protein interaction network prediction. Green nodes denote downregulated hub proteins and red nodes denote upregulated hub proteins. Blue squares represent drugs that act on only one hub protein, whereas purple squares represent drugs that act on two hub proteins.

To further investigate the potential therapeutic drugs for IPF, we performed target drug prediction of hub proteins through the DGIdb; the results are presented in a protein–drug interaction network. As illustrated in Figure 7, 67 possible pharmaceutical agents were ascertained for the five hub proteins. HBEGF, ERBB3, ANGPT2, HGF, and FGF2 were associated with 3, 30, 4, 12, and 21 potential target drugs, respectively. The specific details of these candidate drugs are presented in additional file 4 (Table S4).

A literature search was performed to narrow down key drugs, thereby enhancing the clinical value of drug prediction. Of the 67 predicted drugs, 13 have a potential role in treating pulmonary fibrosis. Some of the 13 drugs have been suggested to be protective in in vitro or in vivo models of pulmonary fibrosis, such as atorvastatin, sirolimus, fluvoxamine, imatinib mesylate, resveratrol, and gefitinib. Sirolimus and imatinib have undergone clinical trials to assess their therapeutic efficacy in managing pulmonary fibrosis. The therapeutic effects of some of the potential drugs have been tested, and in vitro or in vivo studies have developed efficient delivery models that reach the lesion directly, such as lenalidomide, curcumin, and trametinib. Others, such as docetaxel and carboplatin, are commonly used in cancer treatment, and their safety and efficacy have resulted in their being used with nintedanib or pirfenidone in the clinical management of patients with lung cancer-associated IPF.  Table 2 outlines the potential protective roles of the 13 key drugs in pulmonary fibrosis treatment.

### 3.8. GEO Database Validation of the Hub Proteins

**Figure 8:**
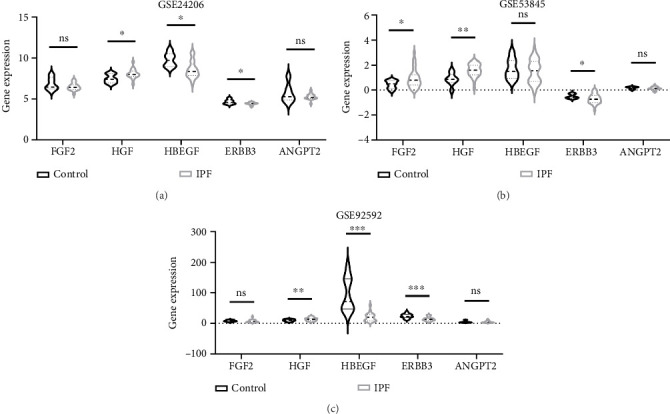
GEO database expression validation of the hub proteins. (a) mRNA expression levels of hub proteins in the GSE24206 dataset; (b) mRNA expression levels of hub proteins in the GSE53845 dataset; (c) mRNA expression levels of hub proteins in the GSE92592 dataset. ^∗^*p* < 0.05; ^∗∗^*p* < 0.01; ^∗∗∗^*p* < 0.001; ns, no significance.

The expression of the identified hub proteins was validated using three independent GEO datasets: GSE24206, GSE53845, and GSE92592. The GSE24206 dataset revealed that the HGF, HBEGF, and ERBB3 mRNA expression patterns in IPF lung tissue versus the control were consistent with the earlier protein microarray results. Nevertheless, FGF2 and ANGPT2 expression was not significantly altered (Figure 8(a)). The GSE53845 dataset demonstrated that the FGF2, HGF, and ERBB3 mRNA expression patterns were aligned with the earlier protein microarray results, while the HBEGF and ANGPT2 expression levels did not change significantly (Figure 8(b)). The expression patterns of the hub proteins in the GSE92592 dataset were consistent with those in the GSE24206 dataset. Specifically, the HGF, HBEGF, and ERBB3 expression trends aligned with those of the present study. Meanwhile, FGF2 and ANGPT2 expression was not significantly different (Figure 8(c)).

### 3.9. Immunohistochemistry (IHC) Validation of the Hub Proteins

**Figure 9:**
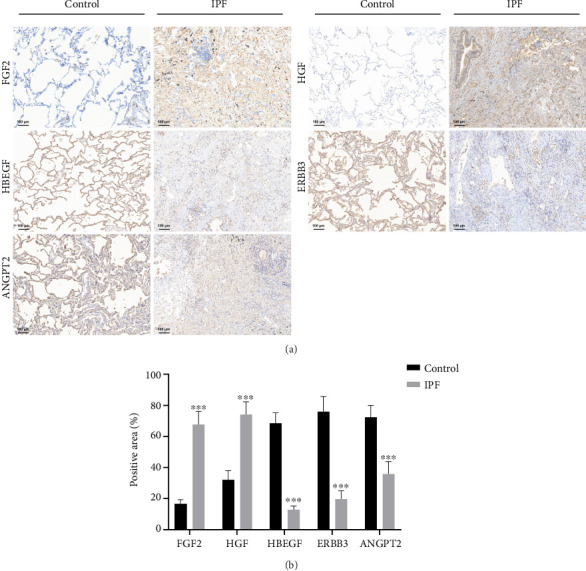
Immunohistochemistry validation of the hub proteins. (a) Immunohistochemical staining of the five hub proteins in the control (*n* = 5) and IPF (*n* = 5) lung tissues (× 100 magnification, scale bars = 100 μm). (b) Quantitative statistical analysis of the immunohistochemistry results. ^∗∗∗^*p* < 0.001.

As transcriptomic data from the GEO database could not fully represent the protein expression, we verified the expression of key cytokines at the protein level using IHC. Consistent with the microarray results, IHC demonstrated that the FGF2 and HGF proteins were generally positively expressed in IPF lungs and weakly or negatively expressed in control lungs, whereas HBEGF, ERBB3, and ANGPT2 were weakly expressed in IPF lungs and positively expressed in control lungs (Figure 9(a)). The observed results were supported by the statistical analysis (Figure 9(b)).

### 3.10. Single-Cell Analysis of Key Cytokines

**Figure 10:**
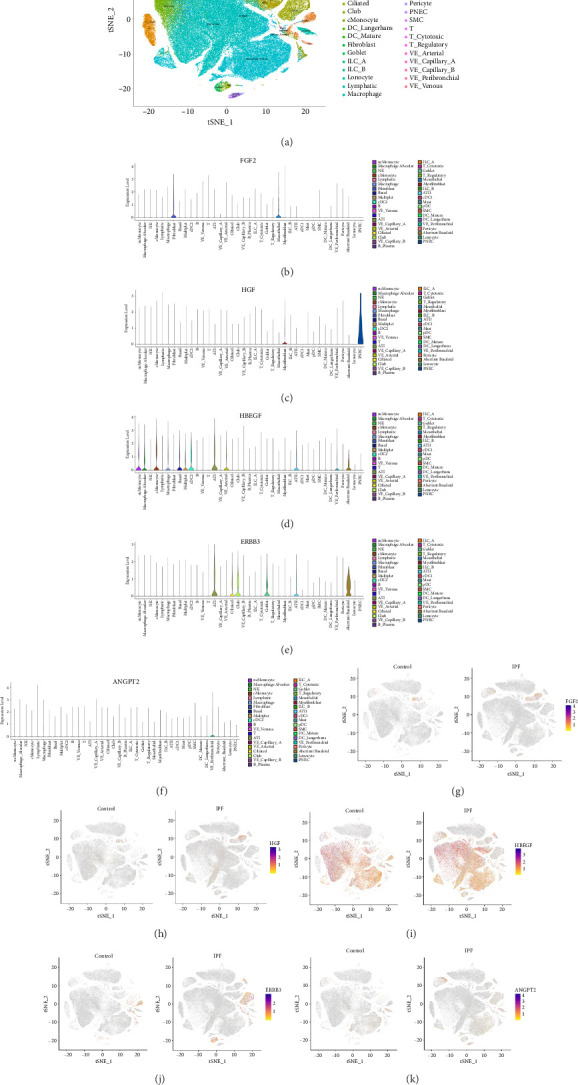
Single-cell analysis reveals major cell types and expression of the key cytokines. (a) t-SNE visualization of the control and IPF lung single-cell populations from GSE136831; (b–f) Violin plots of the expression of (b) FGF2, (c) HGF, (d) HBEGF, (e) ERBB3, and (f) ANGPT2 in each cell population of control and IPF lungs from GSE136831; (g–k) expression distribution of (g) FGF2, (h) HGF, (i) HBEGF, (j) ERBB3, and (k) ANGPT2 in t-SNE plots of control and IPF lungs from GSE136831.

The cellular localization of key proteins was explored by analyzing the single-cell transcriptome data from the GEO dataset GSE136831, which includes 120,066 cells from 32 IPF lungs and 81,538 cells from 28 control donor lungs. As the t-SNE algorithm was performed for dimensionality reduction, high-dimensional data were visualized in a 2D plot and identified 39 distinct cell types (Figure 10(a)). Then, the expression levels of the five key genes in each cell population were analyzed, as were their expression distribution in the control and IPF groups in t-SNE space. Figures 10(b), 10(c), 10(d), 10(e), 10(f), 10(g), 10(h), 10(i), 10(j), 10(k) demonstrate that FGF2 was mainly expressed in fibroblasts, mesothelial cells, and myofibroblasts (Figures 10(b), 10(g)); HGF was primarily expressed in myofibroblasts and pulmonary neuroendocrine cells (PNECs) (Figures 10(c), 10(h)); HBEGF was generally expressed by multiple cell types, including alveolar macrophages, alveolar Type 1 (AT1) cells, alveolar Type 2 (AT2) cells, and aberrant basaloid cells (Figures 10(d), 10(i)); ERBB3 was commonly expressed in ciliated cells, AT1 and AT2 cells, and goblet cells (Figures 10(e), 10(j)); whereas ANGTP2 was predominantly expressed in peribronchial vascular endothelial (pVE) cells (Figures 10(f), 10(k)). The results of the single-cell expression analysis of the hub genes in the control and IPF groups are presented in additional file 5 (Table S5).

## 4. Discussion

IPF is a progressive lung disease where the prognosis is unfavorable, and has no effective pharmacological treatment. It predominantly affects the elderly, and as the global population continues to age, IPF is predicted to impose an increasing physical, psychological, and economic burden on patients and healthcare providers [1, 6]. Although several cytokines, including TGF-β, are pivotal in IPF pathogenesis, its etiology remains unclear. Nevertheless, the link between cytokines and IPF is still a subject of ongoing research. In recent years, numerous studies employing ex vivo and in vivo models of IPF have demonstrated that agents targeting specific cytokines or their receptors have a mitigating effect. Some of these targeting agents, such as pamrevlumab (a fully recombinant anti-CTGF human monoclonal antibody), are already undergoing clinical trials, through which their safety and efficacy are assessed [58–60].

As far as we know, the present study is the first to comprehensively determine the cytokine expression profiles of lung tissues obtained from patients with IPF and healthy donors, using a high-throughput protein microarray based on the principle of double-antibody sandwich method. Following data processing and analysis, a total of 32 DEPs were successfully identified. Among these DEPs, some have previously been reported to be associated with IPF, including CXCL13, MMP7, and FAP. The previously reported expression trends of these DEPs in IPF are consistent with those observed in the present study, and they have been considered as potential blood biomarkers or targets for noninvasive detection on positron emission tomography/computed tomography (PET/CT) imaging to evaluate severity or prognosis in patients with ILD [61–65].

In the present study, the functional enrichment analysis of DEPs facilitated a more in-depth comprehension of the fundamental pathogenesis. The KEGG pathway analysis determined that the DEPs were enriched in several other pathways in addition to the cytokine–cytokine receptor interaction pathway, including the pathways for PI3K–Akt, MAPK, and RAS signaling, proteoglycans in cancer, and EGFR tyrosine kinase inhibitor resistance (Figure 3(b)). The PI3K–Akt and MAPK signaling pathways are two key regulatory pathways in IPF. Recently, focus on the development of novel treatment agents targeting these pathways to alleviate pulmonary fibrosis in ex vivo and in vivo models has increased [3, 66–69]. In addition to the classical IPF regulation pathways, it was discovered that RAS-related signaling—a pathway situated downstream of the endothelial transcription factor FOXF1—is involved in reprogramming normal lung endothelial cells into fibrosis-related endothelial cells in lung fibrosis development [70]. An in vivo model constructed by Luo et al. demonstrated that the Rap1 signaling pathway was involved in the alleviation of bleomycin-induced pulmonary fibrosis by asiaticoside, potentially with the assistance of the adenosine 2A receptor [71]. Furthermore, GSEA of all 440 detected cytokines revealed that they were enriched in various BPs that had not been predicted by the traditional enrichment analyses and included response to insulin, response to peptide hormones, response to nitrogen compounds, and developmental processes involved in reproduction (Figures 3(c), 3(d), 3(e), 3(f), 3(g)). It is noteworthy that most of these processes are consistent with the established risk factors and epidemiological characteristics linked to IPF onset. For example, studies have recently indicated that patients with IPF are often diagnosed with diabetes, suggesting that diabetes is a risk factor for IPF development [72, 73]. This is consistent with the response to insulin item observed in our enrichment results. Similarly, air pollution represents a risk factor for IPF development, and exposure to nitrogen dioxide (NO_2_)—an important component of air pollutants—was positively associated with the risk of mortality in patients with IPF [74, 75]. This is consistent with the response to nitrogen compounds item. Furthermore, epidemiological studies indicate that IPF is a sexually dimorphic disease, occurring predominantly in males, which may be associated with the role of sex-related steroid hormones and their receptors in IPF [76–78]. This finding is in line with the enriched developmental process involved in reproduction in this study. With regard to the item response to peptide hormone, peptide hormones (growth hormone-releasing hormone and relaxin) have been reported to significantly affect the development of IPF. Consequently, intervention in their associated pathways might present a possible avenue for the pharmacological treatment of fibrotic lung disease [79, 80].

Based on the STRING database and the MCC algorithm in Cytoscape, we identified five hub proteins from 32 DEPs, suggesting that they are pivotal in IPF. The association between these hub proteins and IPF has been previously reported, albeit to varying degrees. Some of these hub proteins have been studied more extensively in IPF and are even considered possible biomarkers for IPF diagnosis or prognostic assessment. In contrast, others remain a topic of contention and require further investigation. FGF2 is a potent pro-chemotactic and pro-mitogenic factor that acts through its high-affinity receptors—fibroblast growth factor receptors. FGF2 is involved in diverse BPs, such as cell proliferation, angiogenesis, and wound healing. Previous studies have reported elevated FGF2 expression in the lung tissue and bronchoalveolar lavage fluid from patients with IPF, which is in accordance with the present findings [81, 82]. Sato et al. reported that human fibrocytes facilitate lung fibroblast proliferation by secreting cytokines such as FGF2. They also reported that nintedanib exerted its antifibrotic effect by inhibiting this activity of fibrocytes and reduced the number of fibrocytes accumulated in mouse lungs with lung fibrosis induced by bleomycin [83]. HGF, secreted by mesenchymal cells, is a multifunctional cytokine that acts mainly on epithelial cells and regulates tissue remodeling and angiogenesis. In the pulmonary environment, HGF plays a pivotal role in maintaining alveolar homeostasis and acts as a potent mitogen for epithelial cells in the lower airway [84, 85]. HGF expression was increased in the serum and bronchoalveolar lavage fluid of patients with IPF [84, 86–88]. Consistent with these results, we determined that the IPF lung tissue had higher HGF expression than the control lung tissue. However, the role of HGF in IPF remains controversial. Ex vivo and in vivo studies suggest a potential antifibrotic effect on the basis of the repair and regenerative effects of HGF in injured lung tissue [85, 89, 90]. In contrast, the increased expression of HGF in clinical samples, for example, bronchoalveolar lavage fluid and serum, was positively correlated with IPF severity, suggesting that the potential local protective mechanism of HGF does not effectively prevent the progression of pulmonary fibrosis [84, 86]. HBEGF is a heparin-binding cytokine that exhibits growth factor-promoting activity and exerts a mitogenic effect on fibroblasts. HBEGF is involved in regulating the epidermal growth factor receptor signaling pathway, protein kinase B signaling, and wound healing [91]. The reduced HBEGF expression in IPF lung tissue reported in the present study agrees with its expression pattern in the validation dataset GSE24206. Nevertheless, the precise role of HBEGF in lung fibrosis is unclear. Hult et al. speculated that HBEGF has a pro-fibrotic influence on certain cell types, such as fibroblasts, and an antifibrotic effect on other cell types, such as alveolar epithelial cells [92]. Moreover, An et al. reported that HBEGF inhibits lung fibrosis through its involvement in the regulation of the p38–MAPK pathway and the expression of various inflammatory factors downstream. Furthermore, they proposed that this effect of HBEGF is cell specific [93]. ERBB3, an ERBB family member—also termed human epidermal growth factor receptor 3—is a transmembrane tyrosine kinase receptor. It binds to and is activated by neuregulin-1 and is important in the growth, differentiation, and migration of cells [94]. Numerous tumors exhibit aberrant ERBB3 expression, including lung cancer. Therefore, targeting ERBB3 represents a promising avenue of antitumor therapy against drug resistance [95, 96]. ERBB3 has a less understood role in IPF than other hub proteins. Otsubo et al. used next-generation sequencing and demonstrated that ERBB3 was more frequently altered in the somatic cells of fibrotic lung tissue from patients with IPF-associated lung cancer, which indicates that genetic variants of ERBB3 potentially contribute to IPF development [97]. ANGPT2 is a glycoprotein that affects BPs (vascular development and angiogenesis) by binding to the endothelial TEK receptor tyrosine kinase [98]. ANGPT2 serum levels might be a potential biomarker for evaluating prognosis in patients with acute respiratory distress syndrome [99]. In the present study, ANGPT2 expression was lower in the IPF lung tissue than in the controls. Similarly, Ziora et al. and Uehara et al. noted that ANGPT2 expression was downregulated in the serum of patients with IPF compared to healthy controls. However, these differences were not statistically significant. Furthermore, Uehara et al. stated that serum ANGPT2-level alterations in patients with IPF were associated with disease progression. These findings suggest that ANGPT2 is a valuable biomarker for the assessment of disease prognosis in IPF [100, 101].

The IPF prognosis is poor, and to date, no pharmacological agents can cure it. While pirfenidone and nintedanib (the only antifibrotic drugs currently approved for treating IPF) can slow lung function decline, their efficacy in halting pulmonary fibrosis progression and enhancing patients' quality of life remains limited. Additionally, there are concerns regarding drug resistance and adverse effects [6, 102]. Therefore, safe and effective pharmacological agents are urgently needed. In the present study, we utilized the DGIdb database and a literature search to predict drugs that target key proteins, and identified 13 potential drug candidates. These results provide new insights into the clinical development of drugs for treating IPF (Figure 7, Table S4, Table 2).

Overall, the present study systematically and comprehensively analyzed the cytokine expression profiles in the freshly obtained lung tissues of IPF patients and healthy donors. However, certain study limitations must be noted. First, the high cost of protein microarrays and time constraints meant that only a small cohort was involved, which reduced the statistical power of the study. Subsequent studies can optimize this by expanding the cohort and increasing the sample size. Second, as the CIBERSORT analysis estimates the percentage of different immune cell types in tissues based on gene expression signatures by relying primarily on high-throughput expression profiling data, such as RNA sequencing, the limited proteomic data generated from only 440 proteins by our microarray could have introduced bias. Further studies can address this limitation by increasing detection throughput, expanding sample size, and incorporating flow cytometry to validate the CIBERSORT results. Another significant limitation is that protein expression was evaluated in tissue samples, which have limited clinical applicability. In clinical practice, the histopathological analysis of biopsies is generally sufficient for diagnosing IPF. However, as cytokines are secreted proteins, further analysis of the serum levels of key cytokines can be used to determine their potential as noninvasive biomarkers for diagnosis or prognosis, thereby enhancing their clinical relevance.

## 5. Conclusions

We used protein microarrays to investigate the cytokine expression profiles in IPF and control lung tissues. We identified and functionally analyzed 32 DEPs. We constructed a PPI network for the DEPs and identified five hub proteins, namely, FGF2, HGF, HBEGF, ERBB3, and ANGPT2. The hub proteins were subjected to further analyses, such as immune cell infiltration and potential drug prediction. Finally, the hub protein expression was substantiated using the GEO database. The next stage will be to validate the key cytokines by increasing sample size and sample diversity (e.g., by adding clinical serum samples). In addition, further experiments will be conducted to validate the effects of the drug candidates on the IPF model and explore the underlying mechanisms.

## Figures and Tables

**Table 1:** Clinical data of patients with idiopathic pulmonary fibrosis and control individuals.

Patient ID	Age	Sex	BMI (kg/m^2^)	Former smoker	FVC (%)	DLCO (%)	6MWT (m)
IPF-1	52	Male	22.2	No	67	22	270
IPF-2	65	Male	21.3	Yes	58	10	48
IPF-3	67	Male	24.8	Yes	65	10	203
IPF-4	69	Female	24.7	No	25	38	180
IPF-5	61	Male	17.4	Yes	59	8	184
Control-1	29	Male	20.8	N/A	N/A	N/A	N/A
Control-2	33	Male	18.4	N/A	N/A	N/A	N/A
Control-3	23	Male	21.2	N/A	N/A	N/A	N/A
Control-4	52	Female	21.2	N/A	N/A	N/A	N/A
Control-5	58	Male	24.5	N/A	N/A	N/A	N/A

*Note:* DLCO, diffusing capacity of the lung for carbon monoxide.

Abbreviations: 6MWT, 6-minute walk test; BMI, body mass index; FVC, forced vital capacity.

**Table 2:** Potential protective effects of the identified drugs in pulmonary fibrosis and their potential roles in clinical translational application.

Predicted drug	Description	Potential protective effect in pulmonary fibrosis (clinical/preclinical findings)	References
Atorvastatin	Lipid-lowering drug	Attenuates pulmonary fibrosis in vitro and in vivo via multiple mechanisms, such as regulating myofibroblast differentiation and apoptosis, and suppressing iNOS expression.	[30, 31]
Lenalidomide	Immunomodulatory agent	Inhalation administration in a liposome-loaded form was effective in alleviating bleomycin-induced lung fibrosis in a murine model.	[32, 33]
Sirolimus	Immunosuppressive agent, also known as rapamycin	Exhibited anti-inflammatory and antifibrotic effects on bleomycin-induced pulmonary fibrosis in rats. Short-term treatment reduced circulating fibrocyte concentrations in IPF participants.	[34, 35]
Fluvoxamine	Antidepressant	Alleviated bleomycin-induced lung fibrosis by regulating the cGAS–STING pathway.	[36]
Aspirin	Anti-inflammatory and antiplatelet agent	Alleviated lung fibrosis through the autophagy pathway. Certain aspirin-triggered lipid mediators had antifibrotic effects in mouse lungs with bleomycin-induced pulmonary fibrosis.	[37–40]
Thalidomide	Immunomodulatory agent	Inhibited bleomycin-induced pulmonary fibrosis in mice due to its anti-inflammatory and antioxidant properties. Relieved cough in patients with IPF in small clinical trials.	[41–44]
Imatinib mesylate	Antitumor drug	Had antifibrotic properties. Clinical trials have been conducted to investigate its safety and clinical effects in IPF patients.	[45–47]
Resveratrol	Antiviral and antioxidant agent	Ameliorated in vitro and in vivo pulmonary fibrosis through multiple mechanisms, such as suppressing oxidative stress and regulating the TGF-β–Smad–ERK signaling pathway.	[48, 49]
Docetaxel	Taxoid antineoplastic agent	Used alongside nintedanib for the clinical management of patients with lung cancer-associated IPF.	[50, 51]
Gefitinib	Chemotherapy medication for cancer	Targeted EGFR to attenuate bleomycin- or silica-induced pulmonary fibrosis in murine models.	[52, 53]
Curcumin	Highly pleiotropic molecule	Inhalation of curcumin-loaded large, porous microparticles effectively inhibited bleomycin-induced pulmonary fibrosis in rats.	[54]
Carboplatin	Medication used to treat ovarian cancer	Used with nintedanib or pirfenidone in the clinical management of patients with lung cancer-associated IPF, given its safety and efficacy.	[55, 56]
Trametinib	Antitumor agent	Could be loaded into surface-engineered nanoparticles, which then adhered to monocyte-derived multipotent cells to provide a programmed therapeutic approach for reversing pulmonary fibrosis at the cellular level.	[57]

## Data Availability

The original results are included in the article and its supplementary information files. Queries can be addressed to the corresponding author.

## Supporting Information

Supporting Information 1 Additional file 1 (Table S1.docx): Differentially expressed proteins (DEPs) between the idiopathic pulmonary fibrosis (IPF) and control groups.

Supporting Information 2 Additional file 2 (Table S2.docx): Gene Ontology (GO) and Kyoto Encyclopedia of Genes and Genomes (KEGG) enrichment analyses of differentially expressed proteins.

Supporting Information 3 Additional file 3 (Table S3.xlsx): Gene set enrichment analysis of the microarray expression profile.

Supporting Information 4 Additional file 4 (Table S4.docx): Drugs predicted to interact with the hub proteins according to the DGIdb database.

Supporting Information 5 Additional file 5 (Table S5.xlsx): Single-cell expression analysis of the hub genes in the control and IPF groups.
