# Supplementary material for: Cytokine Expression Profiling in Idiopathic Pulmonary Fibrosis: Insights From Integrative Proteomic Analysis
Source: Can Respir J. 2025 Nov 7;2025:2272156. doi: 10.1155/carj/2272156 (PMC12618133; doi:10.1155/carj/2272156)
Supplement: Supporting Information 1 — Additional file 1 (Table S1.docx): Differentially expressed proteins (DEPs) between the idiopathic pulmonary fibrosis (IPF) and control groups. [file 2272156.f1.docx]

**Table S1** Differentially expressed proteins (DEPs) between the idiopathic pulmonary fibrosis (IPF) and control groups

| Protein name | Fold change | Adjusted *P*-value |
| --- | --- | --- |
| Up-regulated | | |
| C-X-C Motif Chemokine Ligand 13 (CXCL13) | 14.64 | 0.0010 |
| Fibroblast Growth Factor 2 (FGF2) | 7.71 | 0.0056 |
| Cartilage Oligomeric Matrix Protein (COMP) | 7.51 | 0.0106 |
| Complement C5 (C5) | 6.12 | 0.0165 |
| Midkine (MDK) | 5.15 | 0.0244 |
| Fibroblast Activation Protein Alpha (FAP) | 4.64 | 0.0029 |
| Galectin 7 (LGALS7) | 4.06 | 0.0449 |
| Matrix Metallopeptidase 7 (MMP7) | 3.31 | 0.0010 |
| Cadherin 3 (CDH3) | 2.81 | 0.0049 |
| Hepatocyte Growth Factor (HGF) | 2.46 | 0.0380 |
| CD27 Molecule (CD27) | 1.83 | 0.0130 |
| Down-regulated | | |
| CEA Cell Adhesion Molecule 5 (CEACAM5) | 0.08 | 0.0049 |
| Erb-B2 Receptor Tyrosine Kinase 3 (ERBB3) | 0.09 | 0.0033 |
| TNF Superfamily Member 10 (TNFSF10) | 0.13 | 0.0041 |
| Membrane Metalloendopeptidase (MME) | 0.17 | 0.0041 |
| Interleukin 18 Receptor 1 (IL18R1) | 0.22 | 0.0182 |
| Angiopoietin 2 (ANGPT2) | 0.24 | 0.0107 |
| Interleukin 5 Receptor Subunit Alpha (IL5RA) | 0.26 | 0.0061 |
| Epithelial Cell Adhesion Molecule (EPCAM) | 0.30 | 0.0394 |
| Transforming Growth Factor Beta Receptor 3 (TGFBR3) | 0.30 | 0.0275 |
| Insulin Like Growth Factor 1 Receptor (IGF1R) | 0.33 | 0.0436 |
| TYRO3 Protein Tyrosine Kinase (TYRO3) | 0.34 | 0.0141 |
| Kinase Insert Domain Receptor (KDR) | 0.35 | 0.0171 |
| Colony Stimulating Factor 3 Receptor (CSF3R) | 0.36 | 0.0436 |
| Platelet Derived Growth Factor Subunit B (PDGFB) | 0.37 | 0.0449 |
| Interleukin 36 Alpha (IL36A) | 0.37 | 0.0447 |
| Intercellular Adhesion Molecule 2 (ICAM2) | 0.37 | 0.0010 |
| Fas Cell Surface Death Receptor (FAS) | 0.39 | 0.0122 |
| Syndecan 4 (SDC4) | 0.50 | 0.0066 |
| Insulin Like Growth Factor 2 (IGF2) | 0.53 | 0.0196 |
| Interleukin 6 Receptor (IL6R) | 0.55 | 0.0444 |
| Heparin Binding EGF Like Growth Factor (HBEGF) | 0.69 | 0.0291 |
