# Supplementary material for: Cytokine Expression Profiling in Idiopathic Pulmonary Fibrosis: Insights From Integrative Proteomic Analysis
Source: Can Respir J. 2025 Nov 7;2025:2272156. doi: 10.1155/carj/2272156 (PMC12618133; doi:10.1155/carj/2272156)
Supplement: Supporting Information 2 — Additional file 2 (Table S2.docx): Gene Ontology (GO) and Kyoto Encyclopedia of Genes and Genomes (KEGG) enrichment analyses of differentially expressed proteins. [file 2272156.f2.docx]

**Table S2** Gene ontology (GO) and Kyoto Encyclopedia of Genes and Genomes (KEGG) enrichment analyses of differentially expressed proteins

| **Ontology** | **ID** | **Description** | **GeneRatio** | ***P* value** | ***P*.adj** |
| --- | --- | --- | --- | --- | --- |
| **BP** | GO:0060326 | Cell chemotaxis | 10/31 | 5E-11 | 7.3E-08 |
| **BP** | GO:0033674 | Positive regulation of kinase activity | 11/31 | 1E-10 | 9.7E-08 |
| **BP** | GO:0018108 | Peptidyl-tyrosine phosphorylation | 10/31 | 3E-10 | 1E-07 |
| **CC** | GO:0009897 | External side of plasma membrane | 7/31 | 6E-06 | 0.00046 |
| **CC** | GO:0045178 | Basal part of cell | 5/31 | 6E-05 | 0.00244 |
| **CC** | GO:0045121 | Membrane raft | 5/31 | 0.0001 | 0.00263 |
| **MF** | GO:0048018 | Receptor ligand activity | 11/31 | 2E-10 | 1.5E-08 |
| **MF** | GO:0030546 | Signaling receptor activator activity | 11/31 | 3E-10 | 1.5E-08 |
| **MF** | GO:0019838 | Growth factor binding | 7/31 | 3E-09 | 8.9E-08 |
| **KEGG** | hsa01521 | EGFR tyrosine kinase inhibitor resistance | 7/26 | 3E-09 | 1.9E-07 |
| **KEGG** | hsa04151 | PI3K-Akt signaling pathway | 11/26 | 4E-09 | 1.9E-07 |
| **KEGG** | hsa05205 | Proteoglycans in cancer | 9/26 | 7E-09 | 2.4E-07 |
| **KEGG** | hsa04010 | MAPK signaling pathway | 9/26 | 2E-07 | 3.4E-06 |
| **KEGG** | hsa04060 | Cytokine–cytokine receptor interaction | 9/26 | 2E-07 | 3.4E-06 |
| **KEGG** | hsa04014 | Ras signaling pathway | 7/26 | 6E-06 | 0.0001 |
| **KEGG** | hsa04015 | Rap1 signaling pathway | 6/26 | 4E-05 | 0.00058 |
| **KEGG** | hsa05218 | Melanoma | 4/26 | 7E-05 | 0.00091 |
| **KEGG** | hsa04640 | Hematopoietic cell lineage | 4/26 | 0.0002 | 0.0026 |
| **KEGG** | hsa04061 | Viral protein interaction with cytokine and cytokine receptor | 4/26 | 0.0003 | 0.0026 |

GO, Gene ontology; KEGG, Kyoto Encyclopedia of Genes and Genomes; BP, biological processes; CC, cellular components; MF, molecular functions
