# Supplementary material for: Cytokine Expression Profiling in Idiopathic Pulmonary Fibrosis: Insights From Integrative Proteomic Analysis
Source: Can Respir J. 2025 Nov 7;2025:2272156. doi: 10.1155/carj/2272156 (PMC12618133; doi:10.1155/carj/2272156)
Supplement: Supporting Information 4 — Additional file 4 (Table S4.docx): Drugs predicted to interact with the hub proteins according to the DGIdb database. [file 2272156.f4.docx]

**Table S4** Drugs predicted to interact with the hub proteins according to the DGIdb database

| Target gene | Potential drugs | Interaction types | Sources | PMIDs |
| --- | --- | --- | --- | --- |
| FGF2 | REBAMIPIDE | unknown | NCI | 8774986 |
| FGF2 | TRIAMCINOLONE | unknown | NCI | 15923512 |
| FGF2 | ATORVASTATIN | unknown | NCI | 12147302 |
| FGF2 | LENALIDOMIDE | unknown | PharmGKB | 28373444 |
| FGF2 | FAMOTIDINE | unknown | NCI | 11943947 |
| FGF2 | CAFFEINE | unknown | NCI | 15738284 |
| FGF2 | SIROLIMUS | other/unknown | TdgClinicalTrial | 12742462 |
| FGF2 | MUPARFOSTAT | inhibitor | ChemblInteractions |  |
| FGF2 | FLUVOXAMINE | unknown | PharmGKB |  |
| FGF2 | SQUALAMINE | unknown | TTD |  |
| FGF2 | ABT-510 | unknown | NCI | 16051960 |
| FGF2 | INDOMETHACIN | unknown | NCI | 9071932 |
| FGF2 | FP-1039 | suppressor | TALC |  |
| FGF2 | QUIZARTINIB | unknown | CIViC | 27671675 |
| FGF2 | THYROTROPIN | unknown | NCI | 7519916 |
| FGF2 | SUCRALFATE | agonist\|inducer | TTD | 7948825\|8578218\|1957124\|1970337\|8578198 |
| FGF2 | ASPIRIN | unknown | NCI | 9071932 |
| FGF2 | VINCRISTINE | unknown | NCI | 8988045 |
| FGF2 | THALIDOMIDE | unknown | PharmGKB | 28373444 |
| FGF2 | PHENYLEPHRINE | unknown | NCI | 15875663 |
| FGF2 | PYRAZOLE | unknown | NCI | 9730230 |
| HGF | IMATINIB MESYLATE | unknown | NCI | 11439348 |
| HGF | THALIDOMIDE | unknown | NCI | 15939924 |
| HGF | ASPIRIN | unknown | NCI | 11981761 |
| HGF | RILOTUMUMAB | antibody\|inhibitor | TALC\|MyCancerGenome\|TdgClinicalTrial\|  ChemblInteractions\|CancerCommons |  |
| HGF | RESVERATROL | unknown | NCI | 15672869 |
| HGF | MP-0250 | unknown | TTD |  |
| HGF | FICLATUZUMAB | antibody\|inhibitor | TALC\|MyCancerGenome\|TdgClinicalTrial\|  ChemblInteractions\|TTD |  |
| HGF | WORTMANNIN | unknown | NCI | 9603913 |
| HGF | STREPTOZOCIN | unknown | NCI | 12403787 |
| HGF | METHOTREXATE | unknown | NCI | 10732770 |
| HGF | EPIGALOCATECHIN GALLATE | unknown | NCI | 16449979 |
| HGF | RESERPINE | unknown | NCI | 16081063 |
| HBEGF | CETUXIMAB | unknown | PharmGKB |  |
| HBEGF | PANITUMUMAB | unknown | PharmGKB |  |
| HBEGF | KHK-2866 | unknown | TTD |  |
| ERBB3 | TRASTUZUMAB | unknown | DoCM\|CIViC\|PharmGKB | 23680147\|25953157\|30071039 |
| ERBB3 | LAPATINIB | unknown | DoCM\|CIViC\|PharmGKB | 23680147\|25953157\|25398453 |
| ERBB3 | PATRITUMAB | inhibitor | ChemblInteractions\|TTD |  |
| ERBB3 | DACOMITINIB | inhibitor | ChemblInteractions\|MyCancerGenome  ClinicalTrial |  |
| ERBB3 | PERTUZUMAB | unknown | DoCM\|CIViC | 23680147\|26206558\|25216528 |
| ERBB3 | MM-121 | antagonist\|antibody | MyCancerGenome\|ClearityFoundation  ClinicalTrial\|ChemblInteractions\|TTD |  |
| ERBB3 | DULIGOTUZUMAB | antibody | MyCancerGenome\|TTD |  |
| ERBB3 | METHYLCURCUMIN | unknown | DTC | 25753330 |
| ERBB3 | AFATINIB | unknown | CIViC | 27044931\|24685132 |
| ERBB3 | DOCETAXEL | unknown | PharmGKB | 30071039 |
| ERBB3 | GEFITINIB | inhibitor | CIViC\|MyCancerGenomeClinicalTrial\|  PharmGKB | 24685132 |
| ERBB3 | SAPITINIB | inhibitor | TALC\|MyCancerGenome\|ChemblInteractio-ns |  |
| ERBB3 | ELGEMTUMAB | antibody | TTD |  |
| ERBB3 | AV-203 | antibody | TALC\|TTD |  |
| ERBB3 | POZIOTINIB | inhibitor | ChemblInteractions\|MyCancerGenome  ClinicalTrial |  |
| ERBB3 | AMG-888 | antibody | TALC\|MyCancerGenome |  |
| ERBB3 | VANDETANIB | inhibitor | ChemblInteractions |  |
| ERBB3 | ISTIRATUMAB | antibody | TTD |  |
| ERBB3 | MM-111 | antibody | MyCancerGenome\|TdgClinicalTrial |  |
| ERBB3 | OSIMERTINIB MESYLATE | inhibitor | ChemblInteractions |  |
| ERBB3 | SELUMETINIB | unknown | CIViC | 25952648\|24685132 |
| ERBB3 | SERIBANTUMAB | antibody | TTD |  |
| ERBB3 | CETUXIMAB | unknown | CIViC | 25520391 |
| ERBB3 | DIMETHYLCURCUMIN | unknown | DTC | 25753330 |
| ERBB3 | ERLOTINIB | unknown | PharmGKB |  |
| ERBB3 | MARGETUXIMAB | unknown | ClearityFoundationClinicalTrial |  |
| ERBB3 | CURCUMIN | unknown | DTC | 25753330 |
| ERBB3 | CARBOPLATIN | unknown | PharmGKB | 30071039 |
| ERBB3 | AC-480 | unknown | TdgClinicalTrial |  |
| ERBB3 | TRAMETINIB | unknown | CIViC | 25952648 |
| ANGPT2 | NESVACUMAB | inhibitor | TdgClinicalTrial\|ChemblInteractions\|TTD |  |
| ANGPT2 | MEDI-3617 | unknown | TTD |  |
| ANGPT2 | TREBANANIB | inhibitor | ChemblInteractions |  |
| ANGPT2 | RIBAVIRIN | unknown | NCI | 16104024 |
